# Fabrication and optimization of a multielectrode microfluidic electrochemical flow cell for fast and dynamic detection of reaction products

**DOI:** 10.1016/j.mex.2024.103096

**Published:** 2024-12-10

**Authors:** Espen Vinge Fanavoll, David A. Harrington, Svein Sunde, Frode Seland

**Affiliations:** a Department of Materials Science and Engineering, Norwegian University of Science and Technology (NTNU), NO-7491, Trondheim, Norway; b Chemistry Department, University of Victoria, Victoria, British Columbia, V8W 2Y2, Canada

**Keywords:** Soft lithography, Microfluidics, Electrochemical cell, Band electrode, Channel electrode, Collection efficiency, Catalyst deposition, Fabrication and optimization of a multielectrode microfluidic electrochemical flow cell

## Abstract

Construction and experimental validation of electrochemical cells with multiple electrodes in a microfluidic channel is described. Details of the fabrication of the electrodes and polydimethylsiloxane channel using soft lithography methods are given. Calibration of the collection efficiencies and transit times between electrodes validate the use of these cells for fast electrochemical detection of soluble species. Mass transit times between two electrodes down to 3 ms with a flow rate of 200 µL min^-1^ are demonstrated. We demonstrate that there is an upper limit in the electrode width to channel height ratio depending on the electrolyte conductivity. A recommendation for the maximum electrode width to channel height ratio is presented. The electrode width is recommended to not exceed four times the height of the channel in 0.1 M H_2_SO_4_. We also demonstrate operating strategies to minimize the impact of oxygen in air, optimization of stepping motor syringe pump parameters, electrolyte switching, and show how to deposit catalyst particles on a channel electrode.

• Fabrication methods are given for all components of microfluidic flow cells with multiple electrodes
• Conditions are given for improved operation, including geometry, pumping, and electrical parameters.

## Graphical abstract

**Figure:**
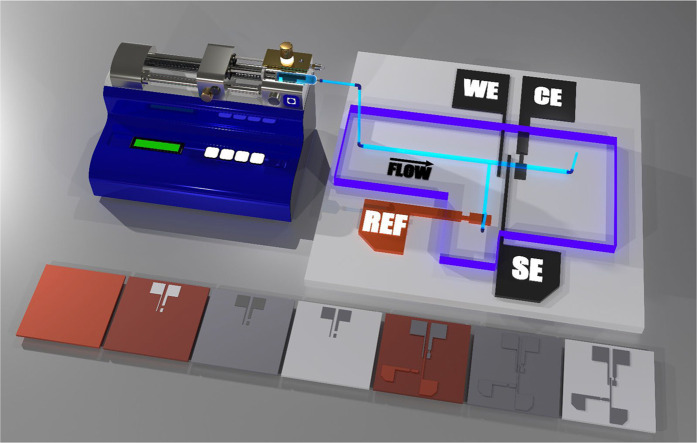

Specifications table

**Table:** 

Subject area:	Chemistry
More specific subject area:	Electroanalytical Chemistry
Name of your method:	Fabrication and optimization of a multielectrode microfluidic electrochemical flow cell
Name and reference of original method:	None
Resource availability:	None

## Background

Microfluidic electrochemical cells in which a solution sequentially flows over several electrodes can be used to detect and characterize soluble intermediates in electrocatalytic reactions, as described in the companion paper [1]. Similar designs have applications in analytical chemistry and lab-on-a-chip reactors. However, production of these devices poses fabrication challenges. Here the fabrication of these cells and some aspects of their optimization is described. The construction and use of on-chip PdH reference electrodes, and control of ionic resistance effects for these devices has already been described [2]. The supplementary information for that work as well as the PhD thesis of Thomas Holm [3] also briefly described the soft-lithography fabrication methods used, but here we give a more complete description, with a number of tips and tradeoffs that we hope will be useful to other researchers.

The validation and optimization of the electrochemical and fluidic performance is described, from methods to reduce oxygen contamination or pumping noise to establishing the agreement of collection efficiency and transit time with theory. Much of this text is adapted from the Ph.D. thesis of Espen Vinge Fanavoll [4]*.*

## Method details

### Design choices and implementation

**Fig. 1:**
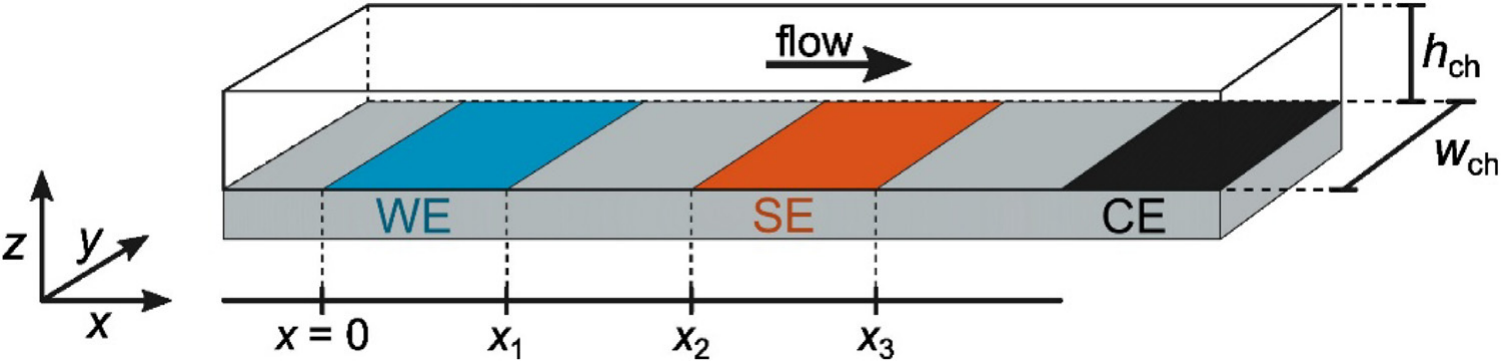
Geometry of the microfluidic electrochemical cell. The length of the electrodes is determined by the width of the microchannel (*w*_ch_, *y*-coordinate). This figure is compressed along the *y*-axis, while the *x* and *z* (channel height, *h*_ch_) axes are to scale for a typical cell geometry.

The class of electrochemical cells described here are microfluidic devices in which an electrolyte solution flows over one or more electrodes, which are used to generate and detect soluble species. The simplest type, Fig. 1, has one working electrode (WE) at which species are generated, a downstream sense electrode (SE) at which detection occurs, a counter electrode (CE) further downstream to carry the current, and an upstream reference electrode (RE) to measure potentials against. The electrodes are deposited on a glass slide, the channel is fabricated from polydimethylsiloxane (PDMS), and then the two are joined.

**Fig. 2:**
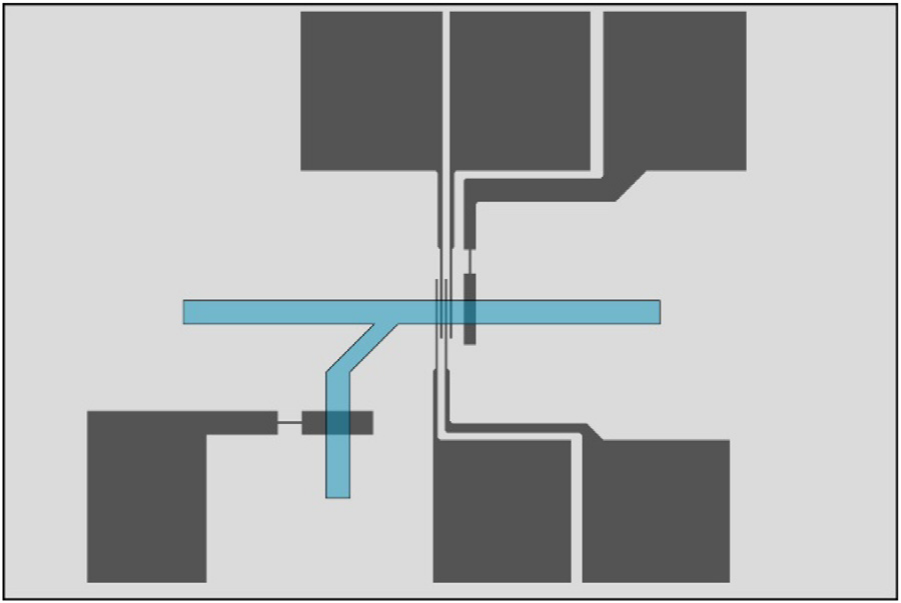
Microfluidic cell design as drawn to scale in AutoCAD. This cell includes four 100 µm working electrodes, a 500 µm counter electrode and a 1 mm reference electrode in a branched channel. Channel width 1 mm.

An example electrode configuration (Fig. 2) used four platinum or palladium microband WE/SE electrodes for flexibility and one CE in a straight channel of height 90 µm or 55µm. The palladium hydride RE was in a side channel where it was uninfluenced by the chemistry of the main channel, and has been described in detail elsewhere [2].

In this work PDMS soft lithography was used to fabricate all the channels. PDMS is simple and relatively inexpensive to prototype and fabricate different channel designs. PDMS is optically transparent, compatible with many chemicals, and the irreversible bond to glass after plasma treatment means that the cell is leak-proof at most applicable flow rates. The cured polymer is flexible, so connection to microbore tubing is normally airtight without further modification.

The main disadvantage of PDMS for electrochemical experiments is the permeability of the polymer. While it is chemically inert to many chemicals, some molecules, especially gases and small organic molecules, are absorbed and may diffuse within the PDMS, to later be released back into the electrolyte. High concentrations of solvents may also cause swelling. It is possible to modify the permeability of PDMS with surface treatment [5]. While the permeability is one of the attractive properties of some applications of microfluidic cells [6], it is definitely a drawback when it comes to using the cells for electroanalytical purposes.

Fabrication of the microfluidic electrochemical cells begins with drawing the cell design in CAD software, such as AutoCAD (Autodesk). Fig. 2 shows an example of a finished microfluidic cell design, including the size of the glass slide and an overlay of the channel. The functional area, where the electrodes intersect with the channel, includes some leeway to make aligning the channel slab and electrode slide easier.

The geometric size of the electrodes are determined by the channel width and the width of the electrode. The lower limit of electrode sizes was found to be mostly determined by the size of defects after fabrication. Electrode widths of 10 µm, or even smaller, are available with acceptable yields. For smaller features, increasing the substrate quality and preventing metal redeposition should be looked into. There is also an upper limit to the electrode sizes that can be functionally used. This is an effect of the solution resistance, and is further discussed in Ref. [2] and in the method validation section. As a rule of thumb, electrode widths should not be larger than 4 times the height of the channel for electrodes used as the working electrode in potentiodynamic experiments.

Away from the channel, the electrode connector may be widened to reduce the ohmic resistance between the electrode and the contact plate. However, wider metal traces under the PDMS can lead to problems with electrolyte leakage, as the internal pressure in the channel may overcome the relatively weak van der Waals forces and allow electrolyte to leak out between the metal and PDMS. One solution to this is to restrict a section of the metal trace close to the functional area of the electrode, as can easily be seen from the reference and counter electrodes in Fig. 2. This significantly increases the pressure required for electrolyte to flow out over those electrodes, while not compromising the conductivity too much. For the cells described here, all electrode traces wider than 100 µm were restricted down to 100µm for 1 mm, and no problems with electrolyte leakage were experienced.

**Fig. 3:**
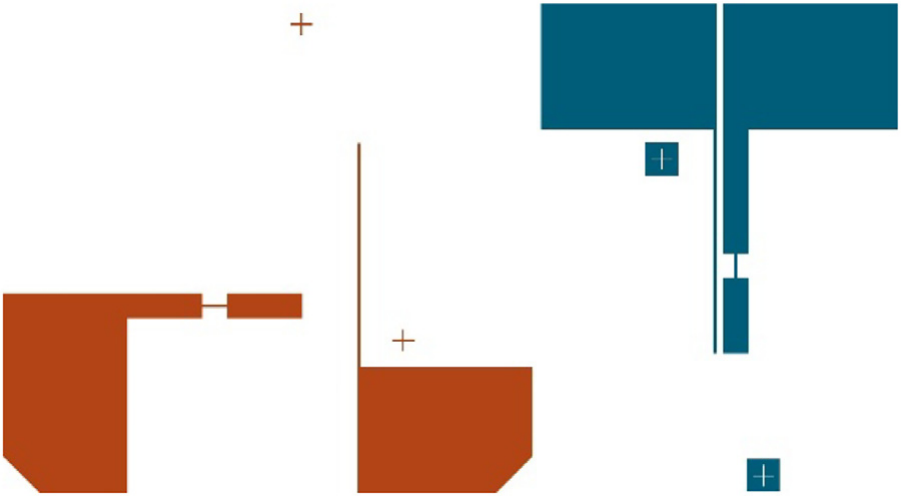
Electrode components on photomask. Blue layer with Pt WE and CE, and red layer with Pd RE and SE. Main channel flow direction to the right.

Fabricating microfluidic electrochemical cells with multiple electrode materials requires some extra consideration in the cell design, as the photolithography and alignment process must be performed several times. When drawing the electrodes for a photomask, the parts must be separated, and alignment crosses are needed to position the electrodes correctly when patterning the electrode slide the second time. An example of this mask design for two electrode materials is shown in Fig. 3. Alignment marks are made by having a target on one layer, and an alignment cross on the other, with a 20 µm gap between the edges of the cross and the target. Alignment marks should be drawn close to, but not obstructing the functional area.

Using the alignment marks, the second layer can be aligned within a few microns of the intended design. A drawback with using photomasks is that there is limited space for different designs on a single mask. Especially when making masks for multiple electrode materials, blank space is needed to prevent overlap between the different designs.

A maskless aligner is an alternative to photomasks, which greatly improves the flexibility in the design of electrode slides. The maskless aligner operates by scanning a laser over the photoresist, turning it on and off rapidly to expose the pattern. To align different layers with the maskless aligner, the different elements need to be in different layers in the same CAD file. The alignment marks are then added by the aligner software, and are automatically detected for the second layer. Using the maskless aligner an assembled microfluidic electrochemical cell can be ready to use a few days after conceiving the device design.

There is no significant limit to how many functional electrodes that can be put in the cell. The main limiting factor is the space for contact plates, which for the designs described here were made to be at least 6 × 6 mm2 to make it easy to solder a wire, leaving room for approximately 10 contact plates. The metal traces on the electrode slide must also make room for the placement of the channel, with some extra room for easier alignment and enough area to have strong PDMS to glass bonding around the channel.

### Fabrication of microfluidic flow cells

The soft lithography methods here are relatively conventional, but reflect specific experience gained while fabricating cells at the NorFab NanoLab cleanroom facility at NTNU Trondheim. The facility includes an ISO 5 grade area with equipment for both photolithography and e-beam lithography, as well as physical vapor deposition of materials by e-beam or sputtering, chemical vapor deposition, reactive ion etching and focused ion beam microscopy.

The cells are fabricated from two components: The electrode slide made by lift-off lithography and physical vapor deposition, and the channel slab made by soft lithography. The instruments and chemicals used in the fabrication are listed in Table 1, Table 2.

**Table 1:** Instruments used in the fabrication of microfluidic flow cells.

Type	Model	Manufacturer
Plasma cleaner	Femto	Diener Electronics
Spin coater		SUSS MicroTec
Mask Aligner	MA6	SUSS MicroTec
Maskless aligner	MLA 150	Heidelberg Instruments
E-beam evaporator	Classic 500	Pfeiffer Vacuum
E-beam evaporator	ATC-2200V	AJA International
Profilometer	Dektak 150	Veeco

**Table 2:** Chemicals used in the fabrication of microfluidic flow cells.

Type	Name	Manufacturer
Photoresist	ma-N 405	micro resist technology
Photoresist	ma-N 440	micro resist technology
Photoresist	SU-8 2100	Microchem
Photoresist	SU-8 3050	Microchem
Photoresist	SU-8 5	Microchem
Developer	ma-D 331/S	micro resist technology
Developer	ma-D 332/S	micro resist technology
Developer	mr-Dev 600	micro resist technology
Remover	mr-Rem 660	micro resist technology
PDMS base	Sylgard 184	Dow Corning
PDMS curing agent	Sylgard 184	Dow Corning

### Electrode fabrication

**Fig. 4:**
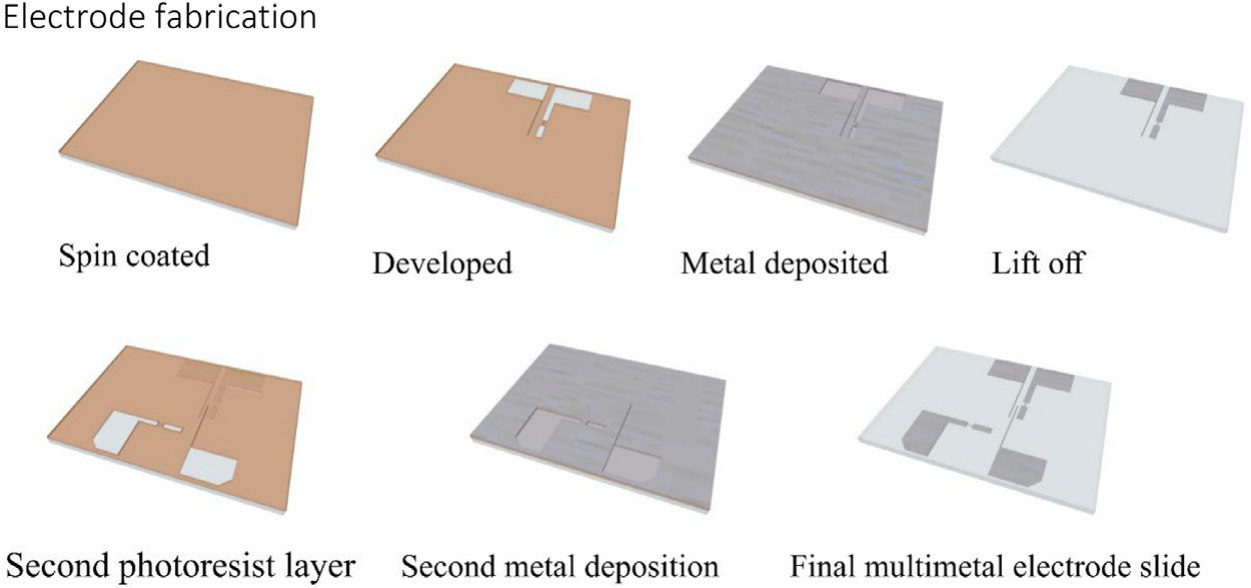
Electrode fabrication steps. Top, left to right: first electrode material (Pt); bottom, left to right: second electrode material (Pd).

The procedure for electrode fabrication is shown schematically in Fig. 4 for two different electrode materials. Standard microscope glass slides (VWR) are used as the substrate for the electrodes. The glass slides are cut in half 25 × 35 mm2 by scoring them across with a diamond scriber, and then snapped cleanly into two pieces, taking care to avoid damaging the glass surface. The glass slides are then loaded into a stainless steel holder and cleaned in acetone in an ultrasonic bath at medium to high power for 10 minutes. After drying off using a nitrogen gun, the ultrasonic cleaning is repeated in isopropanol. Beyond this point it is important to handle the slides with care to avoid contaminating or scratching the glass surface. After drying the slides in nitrogen, the glass slides are further cleaned using oxygen plasma at 50 W for 10 minutes. For an even more thorough cleaning procedure, a piranha etch (H_2_SO_4_ and H_2_O_2_ in a 3:1 ratio) may be used, but this was found to be unnecessary with the cleaning procedure above.

After cleaning, the glass slides are baked at 200 °C for 10 minutes to remove moisture. After cooling down to room temperature, the slides are spin-coated with the negative photoresist. One of two different negative photoresists may be used for the electrodes: ma-N 405 and ma-N 440. These are in the same resist series, but with different viscosities giving different thickness in the developed photoresist. Both are well suited for lift-off electrode fabrication due to good adhesion to glass and good thermal properties. The fabrication parameters for the two resists are listed in Table 3. The ma-N 440 is thicker, and requires longer softbake and a higher exposure dose.

**Table 3:** ma-N photoresist parameters.

Resist	*δ*_resist_	*ω*_spin_	*t*_spin_	*T*_softbake_	*t*_softbake_	Exposure
ma-N 405	0.5 µm	1000 rpm	30 s	100 °C	1 min	400 mJ cm^-2^
ma-N 440	4.1 µm	3000 rpm	30 s	100 °C	4 min	1300 mJ cm^-2^

After spin-coating, the slides are put on a hotplate to softbake the photoresist. After cooling down to room temperature the photoresist was patterned using either a mask aligner or a maskless aligner. The main principle of these is the same: The photoresist is cross-linked by exposure to UV radiation (*λ* = 405 nm), and the pattern decides which parts are not exposed and thus not cross-linked. The cross-linked photoresist becomes darker after the exposure, and the pattern should be clearly visible.

The exposed slides are then put in a beaker with the developer (ma-D 332/S for the ma-N 440 resist). This is perhaps the most critical step, and the main source of low yields and disappointing fabrication results. Underdeveloped photoresist leads to spotty metal deposition, as the metal does not bond properly to the glass. The development time is sensitive to both the strength of the developer and the condition of the photoresist, so the development time stated by the resist manufacturer should be taken as a guideline, not a rule. Slightly different softbake parameters, and also the age of the photoresist may influence the development time. The solution to this is to always monitor the development process. As the non cross-linked photoresist is gradually removed by the developer, the resist thin-film can be monitored by looking at the refraction of light through the film when viewed at the right angle. It is possible to see relatively easily when the last of the resist is removed, leaving clean glass. The resist film is thickest at the edges, and is thus last removed there. Typical development time is around 2 minutes. After the development, the cells should be immediately and carefully rinsed in deionized water for at least 15 seconds, before drying with a nitrogen gun.

Inspection of slides after development used an UV-filtered yellow light microscope. If any underdeveloped photoresist is found, the slide is returned to the developer beaker to finish. Slides with defects, such as broken photoresist, particles or other visible damage to the photoresist are rejected. Before metal deposition, the slides are cleaned in oxygen plasma at 50 W for 5–10 minutes. The slides are mounted to a 4 inch silicon wafer to be loaded into the e-beam evaporator. One wafer has room for 5 electrode slides. A nitrogen gun is used to blow away any potential particles from the glass before deposition.

In the e-beam evaporator, a high voltage (8 kV) electron beam is swept over a source material target to evaporate the target atoms, which are then deposited on the sample (and the walls of the vacuum chamber). The deposition rate is monitored by a quartz crystal microbalance. The metal films are typically deposited at a rate of 5 Å s^-1^, and the sample holder rotated for even deposition. A 10 nm layer of titanium is always deposited first. Titanium provides good adhesion both to the glass, and to the metals deposited on top of it. Alternatively, chrome may be used for the same effect. The adhesion layer is followed by typically 190 nm of the target electrode metal.

After metal deposition the slides are put in a beaker with a solvent-based remover. This removes all the photoresist along with the excess metal on top of it. The removal process takes up to 15 minutes, and is aided by using a small brush. The slides are finished off in clean remover in a separate beaker to make sure all the photoresist and metal particles are removed, and are then rinsed in acetone, dried using nitrogen, and rinsed again in deionized water.

The whole process may be repeated to fabricate electrode slides with multiple electrode materials. As long as the slides are thoroughly cleaned before the second round, no adverse effects were found by performing the lithography process on the previously metallized slides. When the gap between the electrodes was large enough, e.g., when only the reference electrode needs to be palladium, Kapton tape may be used to manually mask off the electrodes, avoiding the second round of photoresist patterning.

**Fig. 5:**
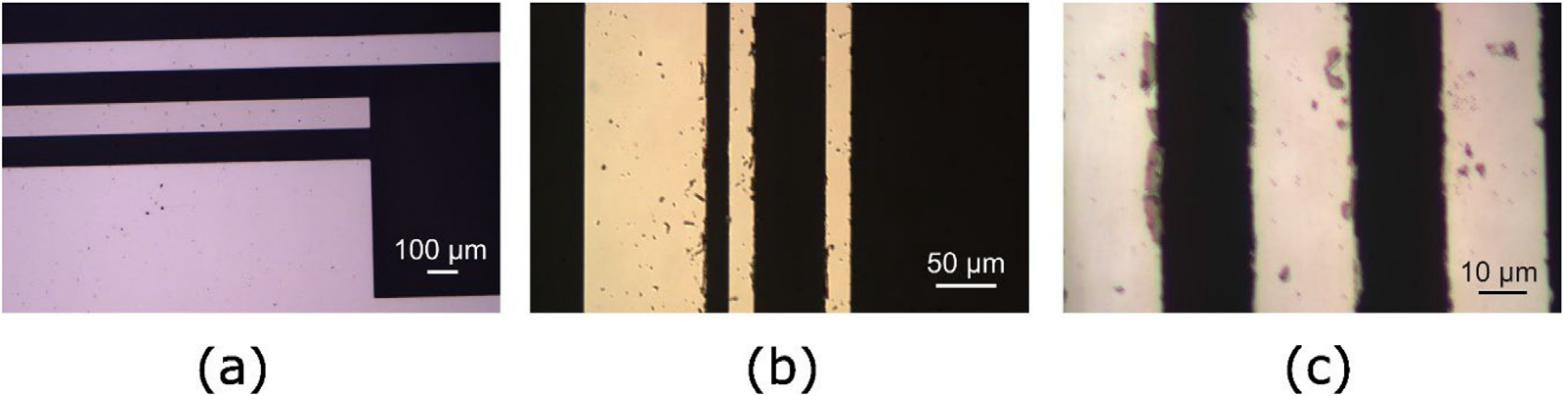
Optical microscope images of finished electrode slides. (a) Typical electrode quality. In this case the top electrode is a 100 µm Pt electrode, and the middle is a 100 µm Pd electrode. (b) Redeposited particles on 20 µm electrodes after lift off process. (c) Metal redeposition on 20 µm Pt electrodes.

All slides should be examined using an optical microscope to check the integrity and cleanness of the electrodes. Fig. 5a shows an optical microscope image of the typical electrode quality of electrode slides used in this work. A relatively common defect is holes in the electrodes, with sizes ranging from submicron to the width of the electrodes. These may stem from particles on the slide prior to metallization, but are more likely due to damage or imperfections in the glass surface. Another common defect is redeposition of removed metal particles on the electrodes. Examples of these can be seen in Fig. 5b and c. Once the electrode slide is dry, these particles become very difficult to remove.

Redeposition of removed metal was found to be especially prominent for gold thin films. One solution to this is to use Scotch^TM^ tape to remove most of the deposited gold film before the chemical lift-off. The gold thin film has stronger adhesion to the tape than the photoresist, and is removed with it, leaving fewer particles in the remover. However, this requires strong film adhesion to the glass, otherwise the electrodes may be damaged as well.

A profilometer may be used to verify the deposited metal thickness, which is typically within 10 % of the target thickness.

Electrode slides of poor electrode quality are unsuitable for electrochemical experiments. The electrodes in Fig. 5b and c are examples of quality below the required standard due to redeposition. A total yield from fabrication of more than 80 % is possible for electrode slides with critical features at 100 µm and above, and of more than 50 % for slides with 20 µm features. The electrode slides are finally cleaned in isopropanol in an ultrasonic bath before assembly.

A commonly used optional step to improve the adhesion between the photoresist and the glass is to use a layer of hexamethyldisilazane (HMDS) as an adsorption promoter. This was found not to be necessary for the photoresists used if the steps above, most notably plasma cleaning and dehydration baking before resist coating, are followed.

### Channel fabrication

The channels are fabricated by casting PDMS over a channel master. A 2 inch silicon wafer (University Wafer) is used as the substrate for the channel master. The wafer is cleaned in acetone and then isopropanol in an ultrasonic bath in the same manner as described for the glass substrates above. The wafer is then cleaned in oxygen plasma at 50 W for 10 minutes and dehydration baked at 200 °C before spin-coating the photoresist. A nitrogen gun is used to blow off any particles.

Three different SU-8 photoresists are used, depending on the channel heights. The fabrication parameters for the different resists to make channel heights (*h*_ch_) are shown in Table 4, Table 5. In general, thicker resist layers require longer baking time and higher exposure doses. The more viscous SU-8 resists need to be spread on the wafer with a plastic spoon in order to be spread properly. It is important to do this carefully to avoid creating too many air bubbles in the resist, though some bubbles are unavoidable with the thick resists. The wafer spin speed is then ramped at 250 rpm/s to 500 rpm and kept for 10 s before the spin speed is increased to the final spin speed, *ω*_spin_, where it is held for *t*_spin_ seconds to obtain the target resist film thickness *δ*_resist_.

**Table 4:** SU-8 pre-exposure parameters.

Resist	*δ*_resist_	*ω*_spin_	*t*_spin_	Softbake			
				*T*_1_	*t*_1_	*T*_2_	*t*_2_
SU-8 2100	90 µm	3000 rpm	30 s	65 °C	5 min	95 °C	25 min
SU-8 3050	55 µm	2500 rpm	40 s	65 °C	5 min	95 °C	15 min
SU-8 5	22 µm	1500 rpm	30 s	65 °C	5 min	95 °C	10 min

**Table 5:** SU-8 post-exposure parameters.

Resist	*δ*_resist_	Exposure	Post-exposure bake				*t*_dev_
			*T*_1_	t_1_	*T*_2_	*t*_2_	
SU-8 2100	90 µm	240 mJ cm^-1^	65 °C	5 min	95 °C	10 min	10 min
SU-8 3050	55 µm	240 mJ cm^-1^	65 °C	5 min	95 °C	10 min	10 min
SU-8 5	22 µm	240 mJ cm^-1^	65 °C	5 min	95 °C	5 min	5 min

If there are too many air bubbles in the resist surface after spin-coating, the wafer is cleaned in developer and the process started again. A few bubbles are acceptable, as long as it is possible to avoid them when aligning the channel master. The coated wafer is put on a hot plate to softbake the photoresist. Thick photoresist layers like SU-8 require more gradual temperature changes, otherwise the resist layer may lose adhesion to the silicon substrate due to thermal shock. The wafer is first baked for 5 minutes at 65 °C before ramping the hot plate up to 95 °C. After the softbake is completed, the hot plate is turned off and the wafer slowly cooled to under 40 °C before taking it off the hot plate. The photomask with the channel is aligned to avoid any air bubbles in the cross-linked resist, and the wafer is then exposed to UV radiation (*λ* = 405 nm).

The exposed pattern is not visible directly after the exposure, but gradually becomes visible during the post-exposure bake. The same principle as for the softbake is followed, starting at 65 °C before ramping up the temperature, and slowly cooling the wafer down again by leaving it on the hot plate to avoid thermal shock. After reaching room temperature, the wafer is developed in the mr-Dev 600 developer, which removes the non-crosslinked photoresist. It is easy to see when the development is finished, leaving a clean Si surface. The wafer is then rinsed in clean developer, followed by acetone and isopropanol before being blown dry with a nitrogen gun.

**Fig. 6:**
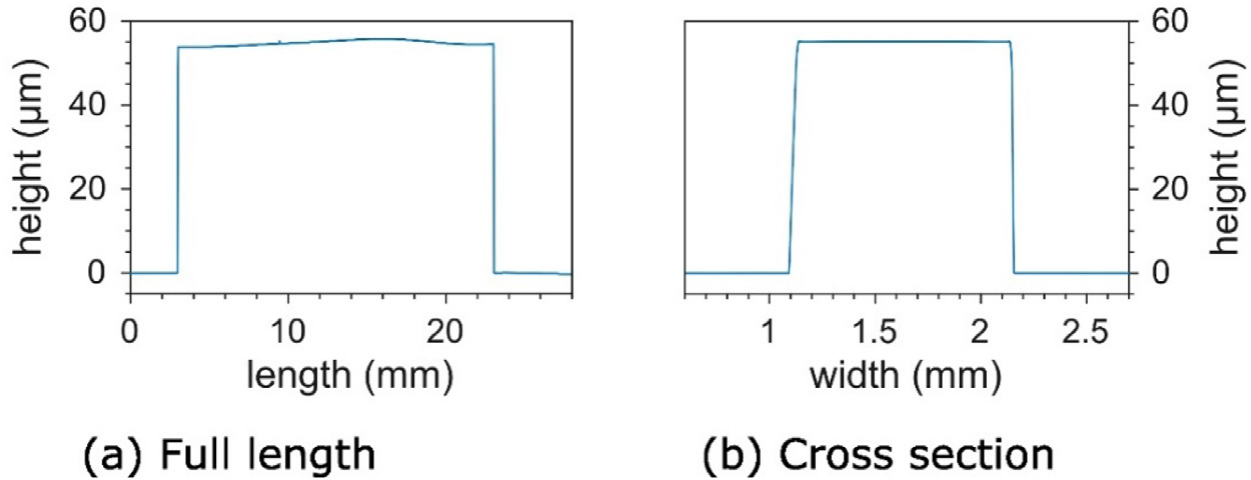
Profilometer measurements of the height of a 55 µm SU-8 channel master: a) full length and b) cross section at where the electrodes will be.

The height and uniformity of the channel master may be checked using a profilometer. Due to evaporation of the photoresist solvent over time, it can be difficult to get the exact resist film thickness from the specifications of the resist manufacturer, so it is necessary to measure this in order to know the actual channel height. As an example, following the specification for 50 µm thickness may give a final resist thickness of 55 µm one time, and 57 µm the next time. Fig. 6 shows the profilometer measurements for the 55 µm channel master. There is less than 2 µm variation in height along the whole 20 mm length of the channel, and the cross section is practically uniform.

An optional step to increase adhesion between the SU-8 and the substrate is to use an adhesion layer of thinner resist, typically SU-8 2, which is softbaked, exposed, and post-exposure baked before spinning the final resist. This was not found to be strictly necessary, as the single layer SU-8/silicon channel masters were mechanically robust and could be used repeatedly without damage.

The channel slab itself is made of PDMS. Uncured PDMS will easily spread and contaminate other samples and equipment, so all PDMS work should be done in a designated fume hood within a cleanroom facility. The polymer base and curing agent are mixed in a 10:1 ratio and stirred for at least 1 minute to homogenize the mixture. The PDMS mixture is then degassed under vacuum for 30 minutes or until most of the air bubbles are gone. About 15 g of the mixture is poured over the 2 inch master wafer. The PDMS is then degassed again for at least 40 minutes to remove all air bubbles before curing at 80 °C for 40 minutes. The cured polymer is cut into the final channel slab, and holes for the electrolyte inlets and outlet are made using a hole punch from the channel side of the slab. The diameter of the hole punch used should be slightly smaller than the outer diameter of the inlet tubing, and the holes made as straight and clean as possible, to make sure that the PDMS and inlet tubing seal properly. For the 1/16 inch (1.59 mm) OD inlet tubing, a 1.25 mm hole punch is suitable.

### Assembly

The finished PDMS slab is thoroughly cleaned of any PDMS or dust particles using Scotch^TM^ tape. Both the electrode slide and the PDMS slab are put in the plasma cleaner for 24 s at 50 W and oxygen flow. This activates the PDMS surface, allowing a strong bond to the glass. The PDMS slab and electrode slide are quickly joined within 1 minute of activation, making sure the channel is placed accurately over the electrodes according to the design. This treatment typically gives a partial irreversible bond between the two parts, and attempting to peel off or realign the channel slab can then rip the PDMS apart leaving pieces attached to the surface. The joined cell is baked at 80 °C for at least 20 minutes to complete the bonding. After the bake, the whole PDMS slab is irreversibly bonded to the glass, and any effort short of treatment with concentrated sulfuric acid does not remove the PDMS from the glass. However, the PDMS did not bond irreversibly to the metal.

The electrical connections to the electrodes are made with tin plated copper wire soldered to the electrode contact plates using lead-free solder. A scalpel blade is used to scratch the surface of the contact plates to improve the wetting between the solder and the electrode metal, and the contact plates are tinned with a small amount of solder before making a solder ball, in order to achieve a durable solder joint. Palladium and gold contact plates are more difficult to solder than palladium as the thin film can quickly alloy with the tin, and care is required not to melt the thin film off the glass slide.

**Fig. 7:**
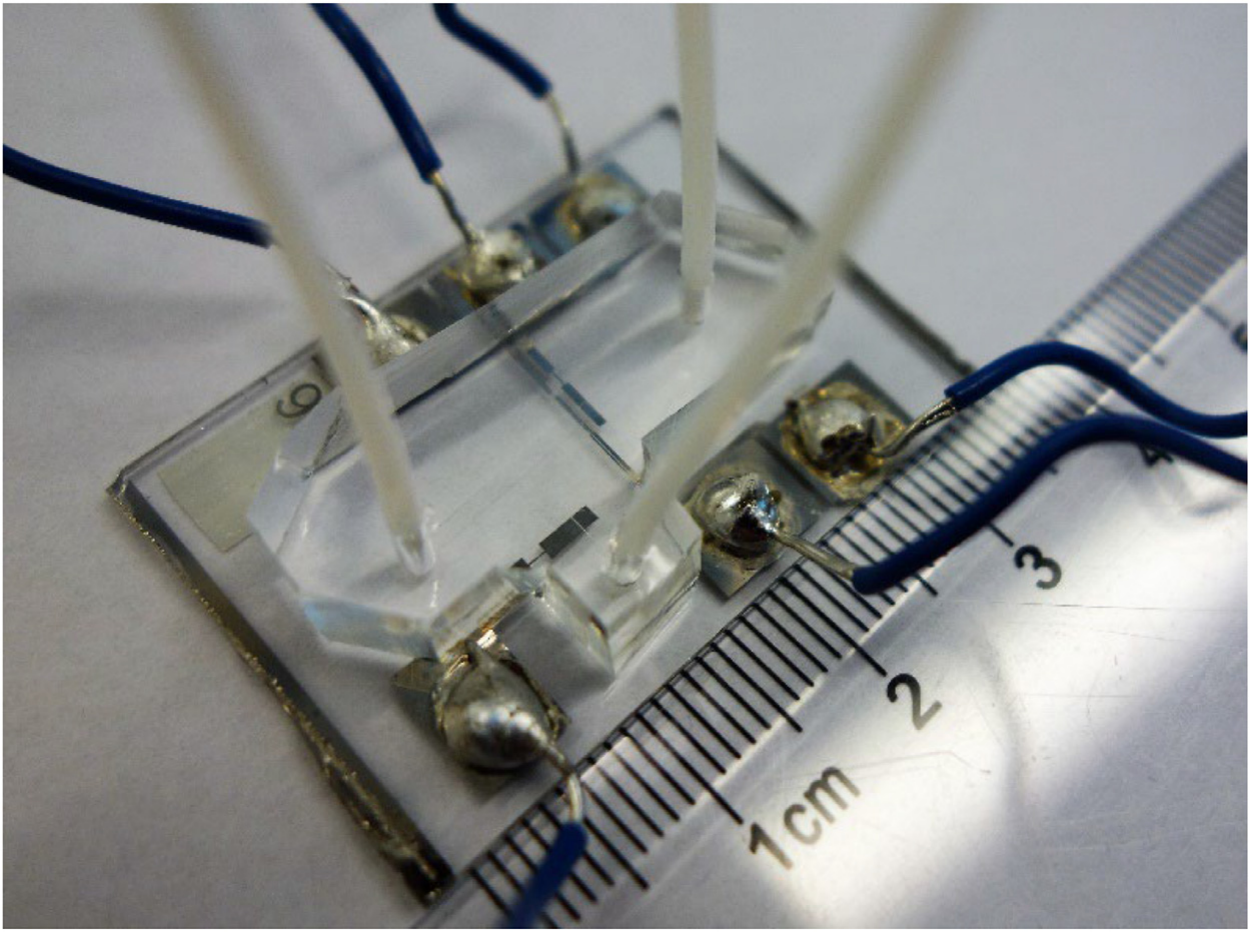
Assembled 6-electrode microfluidic cell. At this scale the four working electrodes (3 × 20 µm and 1 × 100 µm) are not individually distinguishable.

Fluid connections to the microfluidic cell are made with PTFE microbore tubing (e.g. 1/16 inch OD – 1/32 inch ID, Elveflow), which is cut to appropriate lengths with a scalpel blade. It is important to consider the tube size, as it represents almost all of the dead volume in the microfluidic cell. Isopropanol is used to lubricate the insertion, to avoid the PDMS cracking. No silicone was required to seal the connection, as the PDMS forms a good seal with the slightly larger PTFE tubing. The cell is baked at 60 °C to 80 °C to evaporate the isopropanol, and stored before use. The final assembled cell will look something like Fig. 7, depending on the number of electrodes.

**Fig. 8:**
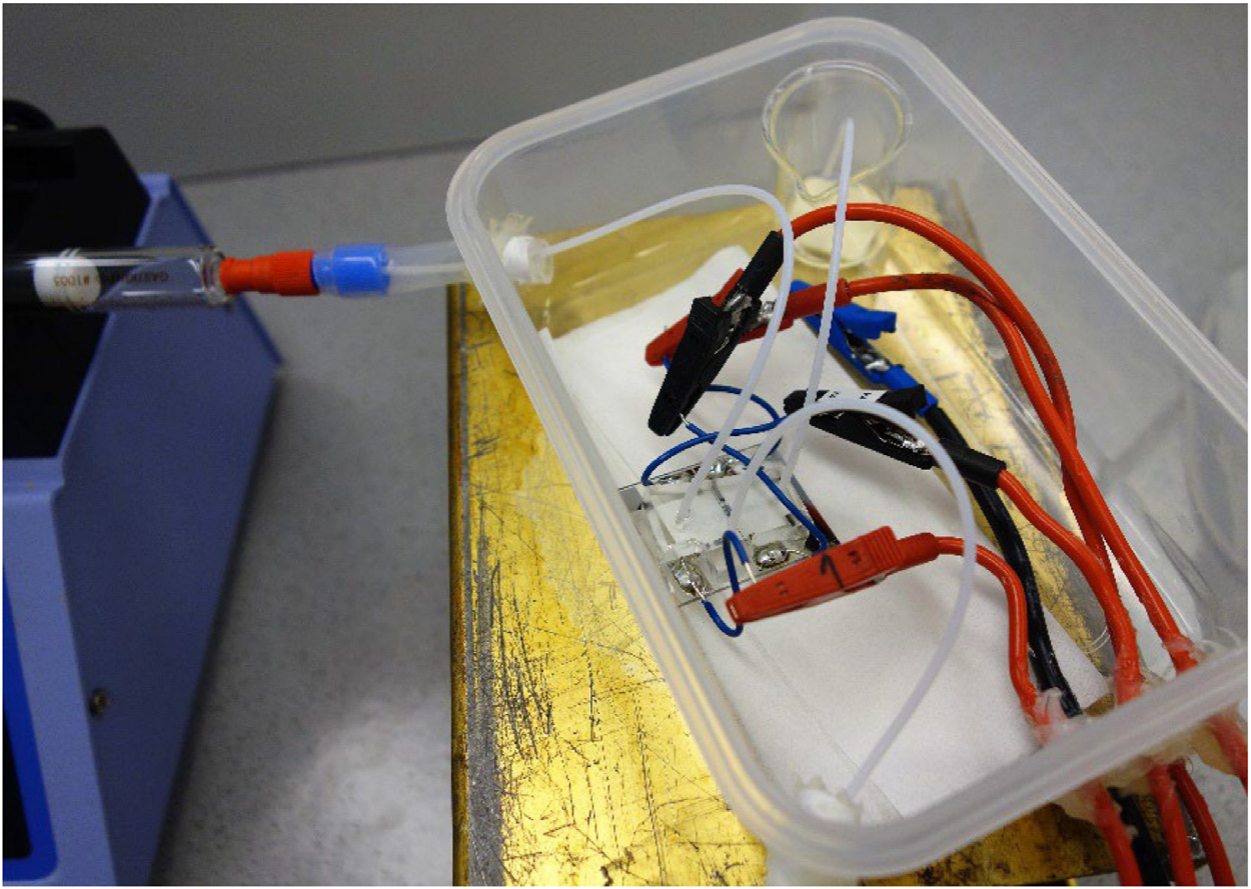
The electrochemical setup for microfluidic cells. The primary inlet is connected to a glass syringe in a syringe pump (left). The inlet tubing is kept inside the purged atmosphere using flexible tubes. Connecting leads to the potentiostats are entering through sealed holes in the box (bottom right). Spent electrolyte is collected in a glass beaker.

In the final electrochemical setup, the primary inlet is connected to a syringe in a programmable syringe pump or pressure pump, while the reference channel inlet is connected to a free syringe. The side channel with the reference electrode was kept stagnant during measurements. The side channel was flushed regularly with supporting electrolyte from the connected syringe. PDMS is oxygen permeable, so it is necessary to keep the microfluidic cell under an inert atmosphere during experiments. A conventional airtight polypropylene/silicone box (∼10 × 15 cm2 and thickness ∼1 mm) was modified to keep the microfluidic electrochemical cell in an oxygen-free environment (see Fig. 8), while still providing electrolyte input and connection to a potentiostat. The cells were mounted and left in the box under inert gas for at least 4 hours, and sometimes overnight, before use to remove oxygen from the PDMS. Either argon or nitrogen gas purging was used, though in this case nitrogen gas may be more effective at purging the oxygen from the cell, as N_2_ is more permeable in PDMS than both O_2_ and Ar [7,8]. The microfluidic flow cells may also be stored in a glove box for a time before use.

Glass syringes with PTFE plungers (Gastight 1000 series LT, Hamilton) were the preferred syringes mostly due to low intrusion of oxygen to the electrolyte over time. Disposable PP/PE syringes (Norm-ject, Air-Tite) were found to slowly allow oxygen into the electrolyte, so it is only advisable to use these for short (< 1 h) experiments, or when oxygen in the electrolyte is not critical.

**Fig. 9:**
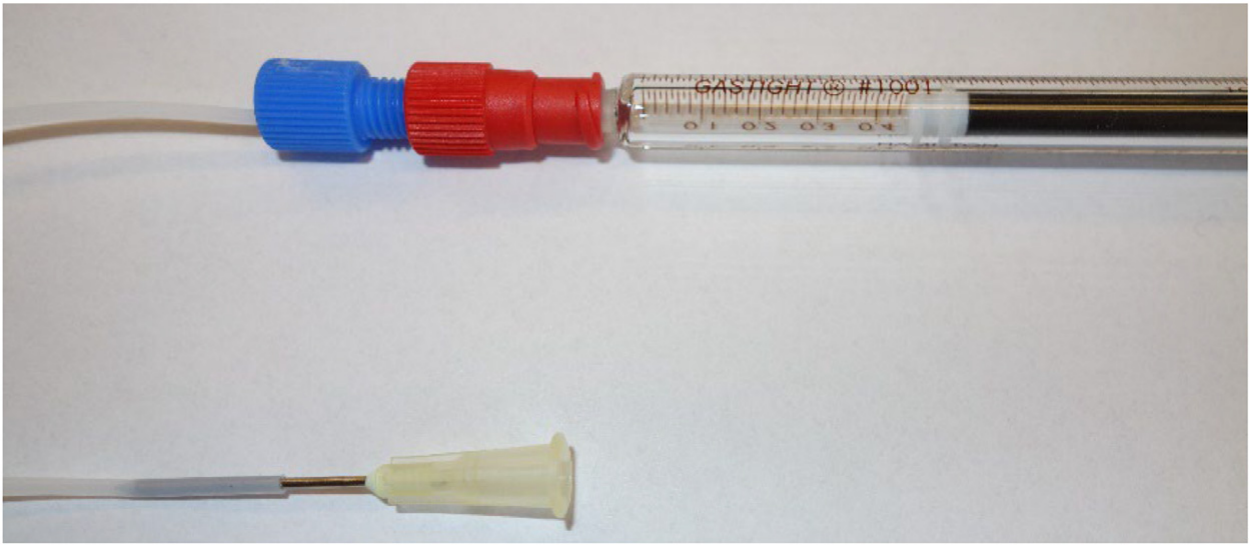
Connecting the microfluidic cell to a syringe. Top: microfluidic connectors. Bottom: disposable needle.

The cell inlets were connected to the microfluidic cells using a setup with a PEEK luer adapter to a PTFE ferrule and end connector (Elveflow), as seen in Fig. 9. Alternatively a disposable stainless steel needle (Air-Tite) with a slightly higher gauge than the tubing ID may be used, though this has a larger dead volume than the microfluidic connectors, as well as an increased risk of needle-stick injuries.

At the end of an experiment, as well as after assembly, the microfluidic cell was cleaned by purging with several milliliters of deionized water, typically at a low flow rate overnight. Purging the cell is especially important when working with acids, as they become more concentrated through evaporation of water, and may damage the PDMS. The cell was then purged with air and baked at 65 °C to 80 °C for at least a few hours or until dry. For a more thorough cleaning, isopropanol or acetone and then isopropanol is used. The cell is then baked and purged with water to remove any trace of the organic solvent.

### Electrochemistry

A BioLogic VMP3 potentiostat was used in all work reported. In addition, a Gamry REF 600 was used for some validation measurements. Experimental measurements of the mass transport limited current, collection efficiency and transit time were performed by applying potential steps (chronoamperometry) to the working electrode. The sense electrode was kept at a constant potential of 1.0 V enabling reduction of $\text{Ru}{\left(\text{bpy}\right)}_{3}^{3+}$ while avoiding oxidization of $\text{Ru}{\left(\text{bpy}\right)}_{3}^{2+}$. The duration of each potential step was at least 15 s, and the reported current was taken to be the average current from the last half of the potential step measurement. The currents were corrected by subtracting the stable background currents measured before and after the potential steps. This background current was negligible at lower flow rates, but more substantial at higher flow rates.

## Method validation

### Validation of electrochemical performance

This section describes experiments to verify that the device behaves quantitatively as predicted by theory, and to determine experimentally the time resolution that is possible. Specifically, the conditions are established for which Poiseuille flow, the 2-D convective-diffusion equations and the standard assumptions of the Lévêque approximation [9] and neglect of axial diffusion are sufficient.

### Collection efficiency

**Fig. 10:**
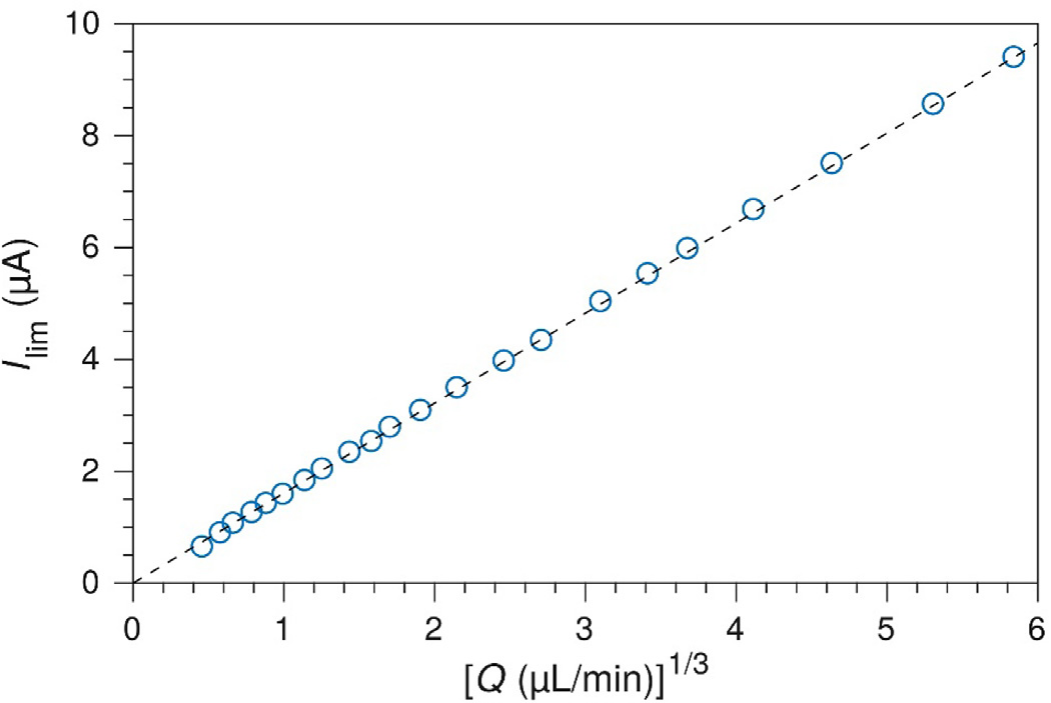
Mass transport limited current. Oxidation current of 5 µM $\text{Ru}{\left(\text{bpy}\right)}_{3}^{2+}$ in 0.1 M H_2_SO_4_ measured at 1.35 V on a Pt electrode. Geometry *h*_ch_ = 55 µm, *w*_ch_ = 1 mm, *w*_el_ = 100 µm.

Under these assumptions, the mass-transport limiting current is given by the Levich Eq. (1) for channel electrodes [10], adapted for the geometry in Fig. 1:

(1) $$I_{\text{lim}}=0.925nFcD^{\frac{2}{3}}Q^{\frac{1}{3}}{\left(\frac{2w_{\text{ch}}x_{1}}{h_{\text{ch}}}\right)}^{\frac{2}{3}}$$

where *n* is the number of electrons transferred in the reaction, *c* is the bulk concentration of the reactant, *D* is the diffusion coefficient, *Q* is the volumetric flow rate, and *x*_1_ is the width of the working electrode in the *x*-direction (see Fig. 1). The prediction that limiting current is proportional to the cube root of the flow rate was confirmed experimentally for a 100 µm wide Pt electrode in a 1 mm x 55 µm microchannel, as seen in Fig. 10. The relationship holds true for all reasonable flow rates, and only breaks down due to depletion at flow rates below 1 µL min^-1^.

The collection efficiency, Eq. (2), gives the fraction of molecules produced at the upstream WE that are detected at the downstream SE under the same assumptions as the Levich equation [11].

(2) $$N=1-F\left(\frac{\alpha }{\beta }\right)+\beta^{\frac{2}{3}}\left(1-F\left(\alpha \right)\right)-{\left(1+\alpha +\beta \right)}^{\frac{2}{3}}\cdot \left[1-F\left(\frac{\alpha }{\beta }\left(1+\alpha +\beta \right)\right)\right]$$

where:

(3) $$F\left(\theta \right)=\frac{3^{\frac{1}{2}}}{4\pi }\text{ln}\left[\frac{{\left(1+\theta^{\frac{1}{3}}\right)}^{3}}{1+\theta }\right]+\frac{3}{2\pi }\text{arctan}\left(\frac{2\theta^{\frac{1}{3}}-1}{3^{\frac{1}{2}}}\right)+\frac{1}{4}$$

and:

(4) $$\alpha =\frac{x_{2}}{x_{1}}-1$$

(5) $$\beta =\frac{x_{3}}{x_{2}}-\frac{x_{2}}{x_{1}}$$

**Fig. 11:**
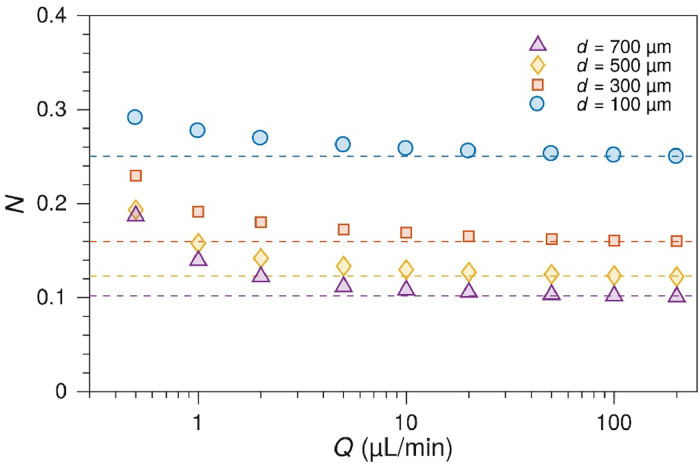
Collection efficiency as a function of the electrode gap. Experimentally measured collection efficiencies between 100 µm Pt electrodes with different electrode gaps (*d* = *x*_2_ - *x*_1_). Dashed lines show the values calculated from Eq. (2). The channel height was 90 µm. The measurements were performed with 1 µm $\text{Ru}{\left(\text{bpy}\right)}_{3}^{2+}$ in a pH 3 phosphate buffer / 0.1 M Na_2_SO_4_ by holding the WE at 1.5 V and the SE at 1.0 V.

**Fig. 12:**
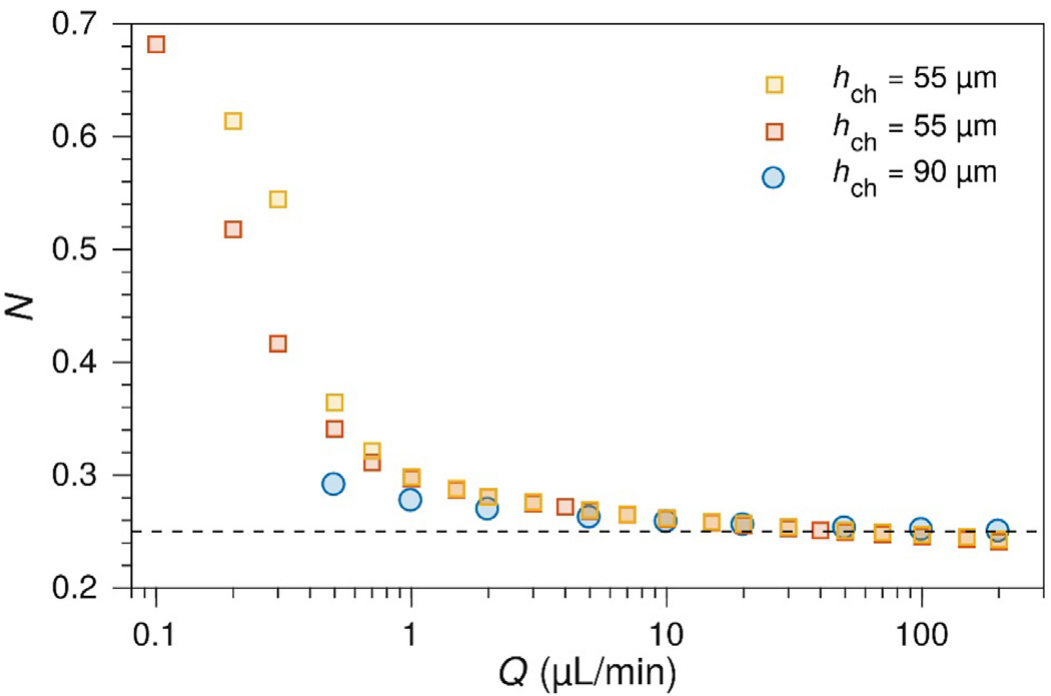
Collection efficiency and channel height. Experimental collection efficiencies for cells with 100 µm wide electrodes with a 100 µm gap, and 90 µm (blue circles) or 55 µm (squares, two replicates) channel heights.

The expressions in Eqs. (4) and (5) are defined by the electrode geometry in Fig. 1. The collection efficiency depends only on the electrode geometry and not the flow rate. However, if the channel height *h*_ch_ is reduced to become comparable to the electrode dimensions, then the Lévêque approximation breaks down and the collection efficiency becomes flow-rate dependent. This effect is seen at slower flow rates in Fig. 11, Fig. 12.

For the typical electrode geometry used here, 100 µm wide electrodes separated by a 100 µm gap, i.e., *x*_1_ = 1, *x*_2_ = 2, *x*_3_ = 3, *N*_∞_ is 0.250. This will be the same for any set of electrodes with the gap the same width as the electrodes.

For thinner channels and lower flow rates, collection efficiencies reach 0.5–0.7 for the standard 100 µm design in a 55 µm high channel at 0.2 µL min^-1^. This is however not an optimal way of increasing the collection efficiency, as the lower flow rates are less stable and harder to work with in practice.

A better way of increasing the collection efficiency is to optimize the electrode geometry. Reducing the upstream electrode width and the electrode gap to 20 µm, with a 100 µm sense electrode, gives a collection efficiency of 0.475 even at higher flow rates Eq. (2).

It should be noted however, that while the collection efficiency is improved with wider sense electrodes, the actual signal-to-noise ratio might suffer. Especially for potentiodynamic techniques, the currents from surface processes such as oxide formation and reduction will scale linearly with the electrode area, while mass transport dependent currents will not. In addition there is a limit to the size of electrodes for small channel heights that can be used in practice for electrochemical measurements in microfludic cells, as will be discussed later in the system performance issues.

### Transit time

**Fig. 13:**
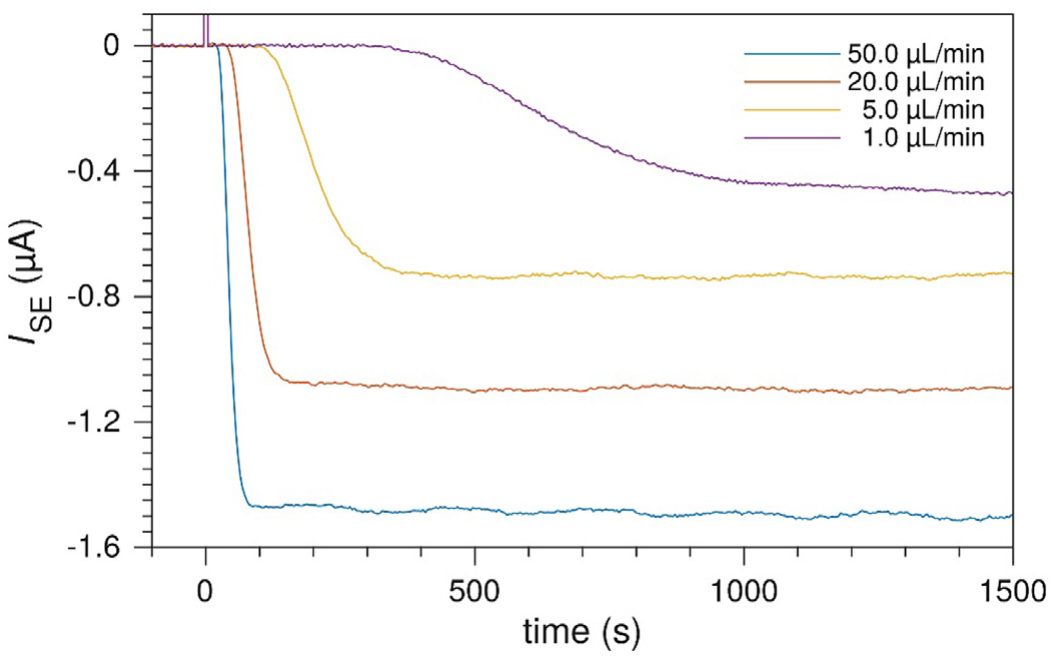
Transit times for 100 µm electrodes and electrode gap at different flow rates. Initial detection of $\text{Ru}{\left(\text{bpy}\right)}_{3}^{3+}$ produced at the upstream WE. Electrode geometry: *x*_1_ = 100 µm, *x*_2_ = 200 µm, *x*_3_ = 300 µm and channel height, *h*_ch_ = 55 µm.

In the multi-electrode microfluidic electrochemical cell, the *transit time* is the approximate time it takes for a certain species produced at the upstream working electrode to be detected at the downstream sense electrode. Fig. 13 shows an example of the detection of $\text{Ru}{\left(\text{bpy}\right)}_{3}^{3+}$ on the SE after a potential step at the upstream WE in 5 mM $\text{Ru}{\left(\text{bpy}\right)}_{3}^{2+}$. The sense electrode detects a brief current spike as the WE potential is stepped at *t* = 0 s. As the electrode gap is on the same scale as the width of the electrodes, there is a significant delay after the initial detection before the steady state collection current. The initial detection time can intuitively be seen as the transport time from *x*_1_ to *x*_2_. The transport involves both downstream convection and diffusion normal to the electrode plane. The transit time does not scale linearly with the flow rate. At 1 µL min^-1^, the transit time coincides with the electrode gap divided by the average velocity.

**Fig. 14:**
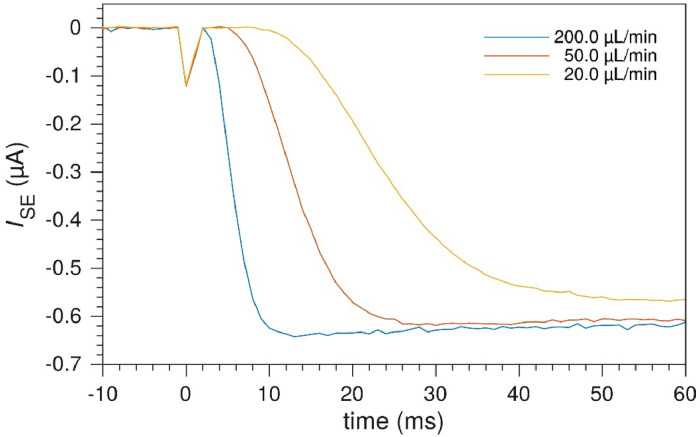
Transit times for 20 µm electrodes and electrode gap at different flow rates. Fast initial detection of $\text{Ru}{\left(\text{bpy}\right)}_{3}^{3+}$ produced at the upstream WE, with a cell geometry optimized towards lower transit times: *x*_1_ = 20 µm, *x*_2_ = 40 µm, *x*_3_ = 60 µm and channel height, *h*_ch_ = 55 µm.

The transit time may be optimized by increasing the flow rate and decreasing the gap between, and the size of, the electrodes. Fig. 14 shows the initial detection in a cell geometry optimized towards lowering the transit time. For 200 µL min^-1^, the initial detection is at 3 ms, and steady state is reached after 12 ms. For reference, the average fluid velocity in the channel at 200 µL min^-1^ is 61 µm ms^-1^.

### System performance issues

This section describes some strategies and variables that affect the performance of the system, other than the fabrication of the devices themselves. It concludes with some tests of electrolyte switching and catalyst deposition that are not fully optimized but are given as proofs of concept.

### Pump performance

For a syringe pump, a syringe acts as the inlet reservoir, and a pusher block on the syringe pump moves the plunger of the syringe. The flow rate is determined by the diameter of the syringe and the speed of the pusher block. One of the major drawbacks with the syringe pump for electrochemical measurements, and mass transport dependent processes in particular, is the nature of the pump's stepping motor. The motor moves in a series of discrete steps and so the syringe plunger is not pushed in a continuous motion. Microstepping is a method of smoothing the motion of the motor [12]. While there is continuous work to improve the stepping angle and the microstepping of these motors, the oscillations they produce in the fluid flow were measurable by the mass transport limited current in the microfluidic electrochemical cells.

**Fig. 15:**
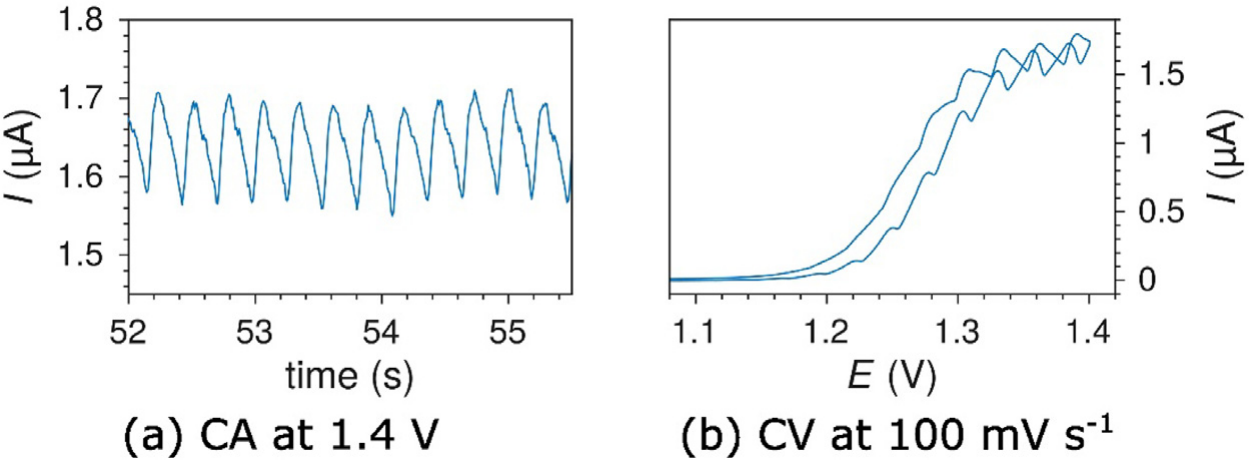
Examples of the oscillations in the measured oxidation current in 5 mM $\text{Ru}{\left(\text{bpy}\right)}_{3}^{2+}$ on a 100 µm wide platinum electrode, using a Harvard Apparatus Model 33 Twin Syringe Pump. The oscillations correspond to the full-step frequency of the syringe pump at this flow rate (3.7 Hz at 5 µL min^-1^).

An example is shown in Fig. 15 for a Model 33 Twin Syringe Pump (Harvard apparatus), which pushes the plunger of the syringe 0.33 µm for each microstep of the motor with 1/4 microstepping. For a typical flow rate (5 µL min^-1^) with a common 1 mL syringe with diameter 4.69 mm, this gives a step frequency of 14.6 Hz for the microsteps and 3.7 Hz for the full steps. These frequencies can be seen in the oscillations in the mass transport limited current, though the microsteps were usually not apparent. The “noisy” flow produces results like those in Fig. 15.

**Fig. 16:**
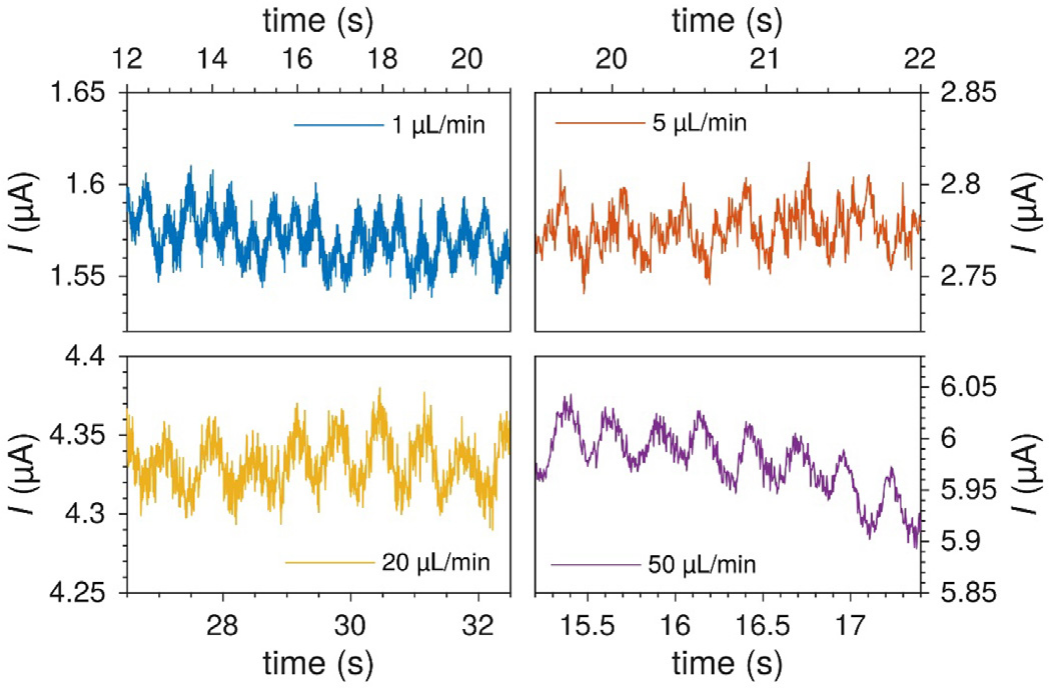
Flow oscillation at various flow rates with a Harvard Apparatus P11 syringe pump and PTFE / glass syringes. Measured in 0.1 M H_2_SO_4_ with 5 mM $\text{Ru}{\left(\text{bpy}\right)}_{3}^{2+}$.

The noise in Fig. 15 is almost 10 % of the mean current, which is detrimental to electrochemical experiments. It should be possible to dampen the oscillations by increasing the compliance of the fluidic circuit, e.g., by introducing a length of Tygon tubing, though this would also decrease the responsivity of the flow. These oscillations are much reduced by using a pump with finer full steps and also 1/16 microstepping, e.g., Pump 11 Pico Plus Elite (P11) (Harvard Apparatus). The pusher block moves 0.031 µm per microstep of the motor. While it is marketed as a virtually stepless syringe pump, some periodic noise is still evident in electrochemical experiments (Fig. 16).

The source of the flow oscillations is easily tracked back to the pump motor as they are periodic with a frequency exactly equal to the step frequency of the motor (*f*_m_) at the given flow rate(*Q*), (*f*_m_ ∝ *Q*). At higher flow rates periodic features with frequencies equal to the step frequency divided by an integer are found. Fig. 16 shows the periodic fluctuations for a selection of flow rates. At 1 µL min^-1^ (*f*_m_ = 2.0 Hz), the periodic features have the same frequency as the pump full steps, though every fourth step looks different. At 5 µL min^-1^ (*f*_m_ = 10.1 Hz) the features with frequency *f*_m_ /4 are more prominent. At higher flow rates periodic features with frequency *f*_m_ /28 are most prominent. The noise levels are typically at 2 % or less. While the noise may not be detrimental to electrochemical experiments, it is useful to know what the source is.

Using high resistance syringe plungers (e.g. PTFE/glass syringes) have the advantage of passively eliminate backflow. The pressure in the main channel can only push a small amount of fluid into the stagnant channels before the pressure is equalized, and the static friction of the syringe prevents further backflow. For example, the side channel with the reference electrode was usually kept stagnant while the main channel was under flow by using the PTFE/glass syringe, while lower resistance plungers (PP/PE or glass/glass) would in some cases be pushed out by the pressure in the channel.

It should be noted that in practice, *completely* stagnant fluid is difficult to achieve in the setup used. Especially when using a pressurized atmosphere to keep oxygen away, small changes in gas pressure as well as ambient pressure may cause the electrolyte to move within the channel, though controllable flow rates should range down to 0.1 µL min^-1^.

A more recent development is the introduction of pressure pumps for microfluidics, using gas pressure over the inlet reservoir to create the pressure required for the flow. This also usually includes a flow meter in a feedback loop, as the fluid flow is not directly known from the applied pressure without complete knowledge of the hydraulic resistance in the circuit as well as outside factors such as the pressure of the purge gas over the outlet reservoir.

A pressure-based pumping system (OB1 MK3, Elveflow) was tested as an alternative to the syringe pump. The advantage of this system is that the gas pressure controlled flow is stepless, and may potentially be tuned to be more stable *and* more responsive than the syringe pump flow. The flow rate is measured by a flow meter between the reservoir and the cell, which is connected in a feedback loop to the pressure controller. The feedback loop must be tuned in a balance between response time and stability. The hydraulic resistance of the fluid circuit must also be tuned to match the pressure range of the controller, which normally involves adding small diameter tubing to the circuit.

For use in the electrochemical setup, the additional components compared to the syringe pump setup proved a challenge. More components exposed to air made it impossible to use this pump in a regular bench setup without introducing too much oxygen to the electrolyte. A functional electrochemical setup with this pump will probably require having the entire fluidic circuit inside a glove box or glove bag to exclude oxygen.

Another practical challenge with the pressure pump system is that it is vulnerable to backflow. When working with multiple inlet cells, keeping one inlet stagnant requires actively measuring the flow rate and keeping it at zero by adjusting the pressure over the inlet reservoir.

If these experimental challenges are solved, the pressure pump system is more flexible than the syringe pump in terms of the flow programs that can be applied, and the increased responsivity may enable more hydrodynamic investigations than are possible with a syringe pump.

### Electrolyte switching

One advantage of microfluidic electrochemical cells is that they allow for fast switching of the electrolyte. One major consideration here is the dead volume of the fluid circuit. With a single inlet the PTFE tubing represents almost all of the dead volume, which is in the order of 0.1 mL for a typical cell used in this work. In addition to this, the flow in the tubing is always laminar, and several times the dead volume is required to completely flush the previous electrolyte. This can of course be reduced by using thinner tubing, but the best way to decrease the dead volume and increase the electrolyte switching capabilities is to use a multiple input cell with a junction in the microchannel. The dead volume is then less than 1 µL.

**Fig. 17:**
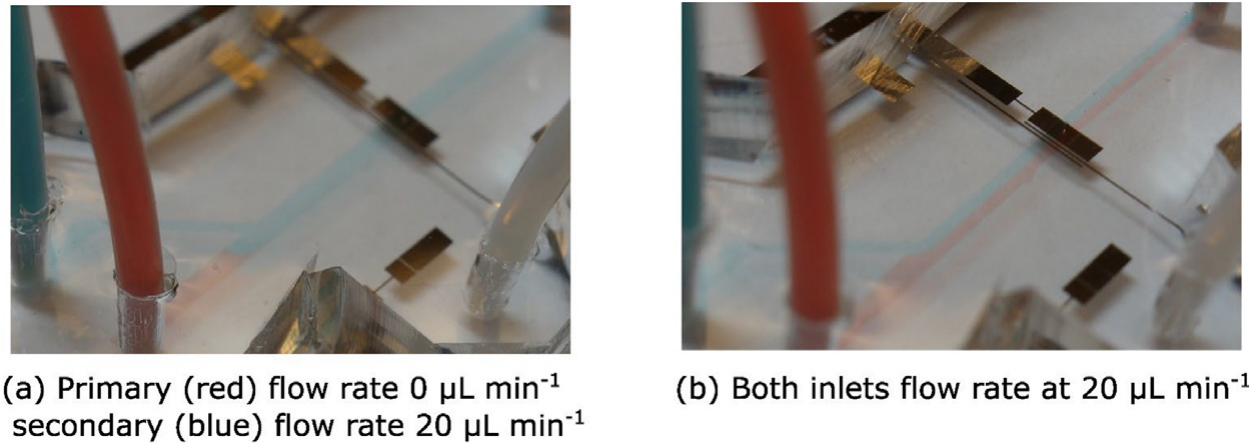
Visualization of electrolyte switching with dyed water. Flow to primary (red, straight) and secondary (blue, branched) inlets was provided by the pressure pump, and the reference channel inlet kept stagnant connected to a PTFE / glass syringe. For scale, the reference electrode in the bottom right of the figure is 1 mm wide.

In Fig. 17, the electrolyte switching and dual flow in a triple inlet microfluidic electrochemical cell is visualized using dyed water. The electrolyte over the working electrode can be switched in less then 1 second. The fluid in the stagnant inlets is pushed slightly back by the fluid under flow. When both main inlets are under flow, a clean dual flow is formed with very low mixing. This mode is used for example in membraneless fuel cells to separate the anode and cathode. Note that the reference side channel (branching down from the main channel towards the reference electrode), which is kept stagnant with clear water using a PTFE / glass syringe, is not much affected by the flow in the main channel.

Another possibility with microfluidic electrochemical cells is to change the concentration by in situ mixing of different electrolytes. The device must then be tailored to allow for complete mixing of the electrolytes [13], which is the opposite case of Fig. 17b. With the pressure pump system, there is also commercial equipment which may be used to handle multiple electrolytes.

### Gas bubbles

**Fig. 18:**
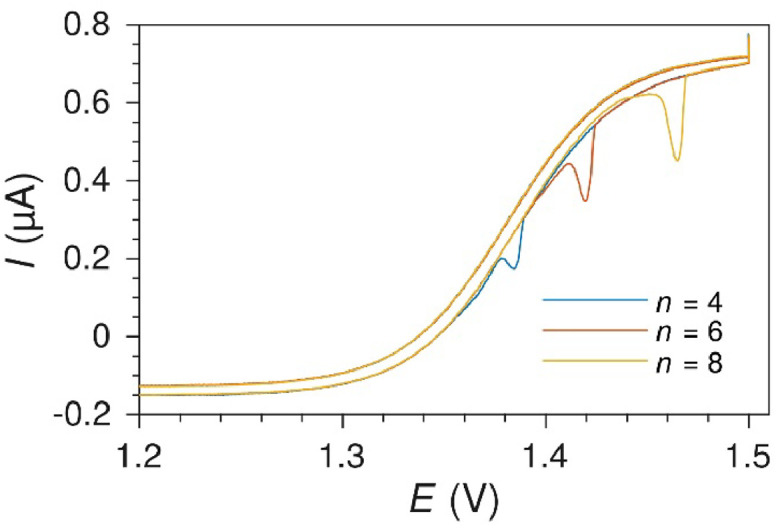
Current drops due to a gas bubble growing and blocking the flow momentarily during cyclic voltammetry on Pt at 100 mV s^-1^ in 1 mM $\text{Ru}{\left(\text{bpy}\right)}_{3}^{2+}$ at 50 µL min^-1^. The current drops are spaced evenly in 20 s intervals.

A common source of flow disturbances not originating from the syringe pump, was gas bubbles in the microchannel. A gas bubble would often grow and be trapped in the intersection between the inlet tubing and the PDMS and momentarily block the electrolyte flow. This blocking was very periodic, and led to characteristic and regular drops in the flow rate and thus also the measured current. The effect was easily observed during cyclic voltammetry measurements with the reversible $\text{Ru}{\left(\text{bpy}\right)}_{3}^{2+}$ species (Fig. 18) showing a periodic drop in current every 20 seconds (i.e. every 2nd cycle). Trapped gas bubbles are relatively easy to remove with high flow rates if they are discovered.

Another problem with gas bubbles is that they may get trapped between the working electrode and the reference electrode, sometimes blocking off the electrical contact between the electrodes. When this happens the potentiostat may increase the current to the electrode in order to achieve the target potential. This actually caused some of the less robust palladium electrodes to be completely dissolved in a matter of seconds. Gas bubbles were especially problematic when working with external reference electrodes, as the bubbles would easily become trapped at the outlet of the cell.

### Electrode size limit

**Fig. 19:**
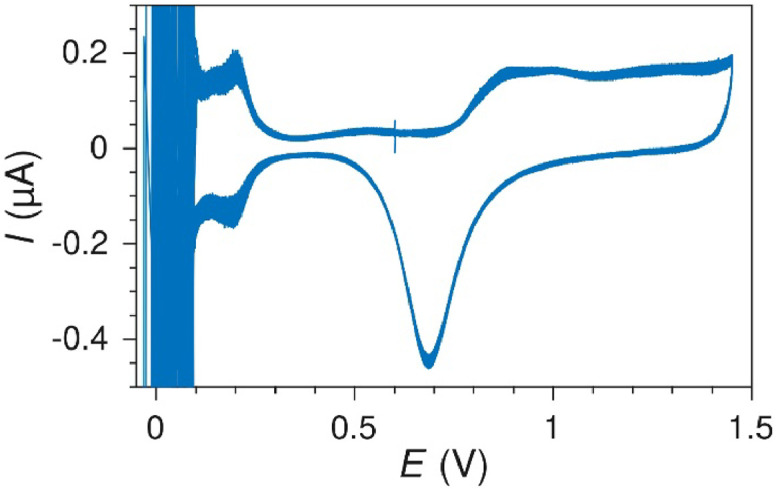
Cyclic voltammograms at a 100 µm wide Pt electrode in a 22 µm high microchannel in 0.1 M H_2_SO_4_ electrolyte. Sweep rate 100 mV s^-1^. Notice the noise below 0.1 V.

An upper limit of the width of the electrodes was found from cyclic voltammetry measurements and depends on electrode and channel geometries. Note that the electrode widths used here are generally larger than the height of the microchannel. For electrodes that are too wide compared to the channel height, the potential distribution over the electrode will be uneven due to the high resistance in the channel. The effect of having a too high electrode width to channel height ratio can be observed during cyclic voltammetry. The effect was first visible at the lowest potential region in the underpotential deposition of hydrogen as oscillating noise with amplitude the same as the current range of the measurement (Fig. 19). For other geometries, the noise would also cover the rest of the hydrogen underpotential deposition region, parts of the oxide region, or the whole voltammogram as well. The effect was the same at multiple scan rates. The voltammograms were otherwise stable, indicating that the effect is not real in terms of electrode surface processes, but rather a problem with the potentiostat control loop due to the current distribution resulting from limited electrolyte conductivity.

The effect was seen both in cells with 100 µm wide electrodes in a 22 µm high channel, and on 500 µm wide electrodes in a ∼100 µm high channel, with both a BioLogic VMP3 and a Gamry Reference 600 potentiostat, and with both the internal PdH and an external RHE reference electrodes. For the latter, this was only observed if the working electrode was situated between the counter electrode and the outlet reservoir containing the RHE reference electrode. The effect was reduced or eliminated entirely when increasing the conductivity of the electrolyte, e.g. by using 0.5 M H_2_SO_4_ instead of 0.1 M H_2_SO_4_. This shows that the effect originates from the limited conductivity of the electrolyte.

As a rule of thumb, the electrode width should not exceed 4 times the height of the channel when using 0.1 M sulfuric acid or similar electrolytes.

### Catalyst deposition

**Fig. 20:**
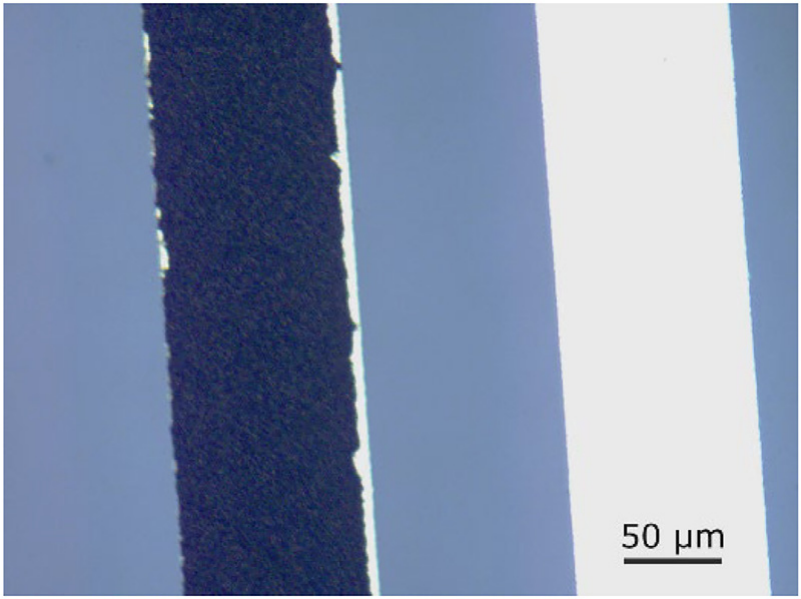
Optical microscope image of a Pt/Vulcan catalyst layer deposited on a 100 µm wide Au electrode (left), next to a clean Au electrode (right).

Catalyst particles can be directly deposited onto the microband electrodes using, for example by drop-casting. A catalyst dispersion was made by sonicating a commercial 60 wt% Pt/Vulcan catalyst in deionized water until the dispersion was stable for at least 30 minutes. A 100 µm wide gold electrode was coated with ma-N 405 photoresist, after which a small section of the electrode was exposed and developed. The slide was cleaned in oxygen plasma before a droplet of the catalyst dispersion was applied evenly to the electrode surface and dried in air. The electrode was again cleaned in oxygen plasma before the whole slide was immersed in a beaker with acetone to remove the photoresist with excess catalyst particles. The photoresist was carefully lifted off as a film, leaving most of the catalyst particles deposited on to the gold electrode still attached. The electrode slide was then carefully rinsed in isopropanol. The catalyst layer was found to almost completely cover the gold, as seen in the microscope image in Fig. 20.

The average thickness of the catalyst layer was found with a profilometer to be around 1 µm. The catalyst layer was found to be robust enough to be used at high flow rates in the microfluidic cell. When tested electrochemically by cyclic voltammetry in 0.1 M H_2_SO_4_, the Pt catalyst showed an electrochemically active area 5 times larger than that of a flat Pt electrode of the same size, with no or little trace of the gold substrate electrode.

This part is included in order to demonstrate the possibility of employing catalyst-covered microband electrodes for microfluidic cells. This method combined with down-stream detection electrodes allow for e.g. measuring current efficiency towards certain byproducts with respect to catalyst composition. Further work is required to obtain a more controlled amount of catalyst, perhaps by self assembly of particles. It may also be possible to make use of different techniques such as screen printing [14,15] or pyrolysis of carbon-based photoresists [[16], [17], [18]] to make a more electrochemically inert carbon substrate electrode for the catalyst.

## Limitations

None.

## Ethics statements

None.

## Supplementary material *and/or* additional information [OPTIONAL]

None.

## CRediT authorship contribution statement

**Espen Vinge Fanavoll:** Writing – review & editing, Writing – original draft, Visualization, Software, Methodology, Investigation, Formal analysis, Data curation, Conceptualization. **David A. Harrington:** Writing – review & editing, Writing – original draft, Validation, Supervision, Resources, Project administration, Methodology, Funding acquisition, Conceptualization. **Svein Sunde:** Writing – review & editing, Writing – original draft, Validation, Supervision, Methodology, Formal analysis, Conceptualization. **Frode Seland:** Writing – review & editing, Writing – original draft, Validation, Supervision, Resources, Project administration, Methodology, Funding acquisition, Formal analysis, Conceptualization.

## Declaration of competing interest

The authors declare that they have no known competing financial interests or personal relationships that could have appeared to influence the work reported in this paper.

## Data Availability

Data will be made available on request.
